# Naprapathy versus orthopaedic standard care for common musculoskeletal disorders: an 8-year follow-up of a pragmatic randomized controlled trial in Sweden

**DOI:** 10.1186/s12998-021-00400-6

**Published:** 2021-11-02

**Authors:** Stina Lilje, Andreas Eklund, Anders Wykman, Tobias Sundberg, Eva Skillgate

**Affiliations:** 1 Musculoskeletal and Sports Injury Epidemiology Center, Department of Health Promotion Science, Sophiahemmet University, Valhallav. 91, 114 28 Stockholm, Sweden; 2 Unit of Intervention and Implementation Research for Worker Health, Institute of Environmental Medicine (IMM), Karolinska Institutet, Nobels v. 13, 171 77 Stockholm, Sweden; 3 Helsingborg Hospital, Lund University, Charlotte Yléns g. 10, 252 23 Lund, Sweden

**Keywords:** Orthopaedic waiting lists, Non-surgical musculoskeletal disorders, Long-term follow-up, Manual therapy, Complementary medicine, Integrative medicine

## Abstract

**Background:**

Musculoskeletal pain is among the most common reasons for seeking care, specialist competence for its treatment in primary care limited and waiting lists for orthopaedics often amongst the longest. Many referrals to orthopaedics do not concern disorders that benefit from surgery. Manual therapy is effective, yet not integrated in national health care systems, and there is a lack of research on other than neck and low back pain, and a lack of long-term follow-ups*.* The present study evaluates the long-term effects of a manual therapy (naprapathy) for common orthopaedic disorders.

**Methods:**

An 8-year follow-up (96 months) of a pragmatic randomized controlled trial of naprapathy (experimental group) versus standard orthopaedic care (control group) for non-surgical patients of working age with the most common musculoskeletal disorders on the waiting lists (n = 78). Bodily pain, physical function (SF36), Quality of life (QoL; SF6D), and data on health care utilization were collected. The treatments lasted from January 2007 to November 2007.

**Results:**

N = 75 participants in the original study sample completed the 8-year follow-up. The differences in bodily pain (21.7 (95% CI: 9.1–34.3)), physical function (17.6 (6.7–28.4)), and QoLs (0.823 (95% CI: 0.785–0.862) compared with 0.713 (95% CI: 0.668–0.758)) were statistically significantly in favor of the experimental group (*p*-values < 0.01). After sensitivity analysis the experimental group had altogether 260 health care visits compared with 1161 in the control group.

**Conclusions:**

Naprapathy is a continuously effective treatment. Together with earlier research our study suggests that specialized manual therapy should be considered when triaging patients with common non-surgical musculoskeletal disorders in national health care systems.

*Trial registration*: Not applicable, as per information given by ClinicalTrials.gov.

## Introduction

Musculoskeletal pain and disorders in the locomotor system are dominant causes for years lived with disability (Disability Adjusted Life Years; DALY), decreased quality of life, and a potential income loss for the individual [1]. Chronic musculoskeletal pain is further associated with increased healthcare seeking behavior, reduced work ability, sick leave, and high socioeconomic costs due to more frequent early retirement [2]. It is also one of the most common reasons for seeking care and constitutes around half of the consultations in primary care [3, 4]. The competence for managing musculoskeletal disorders in primary health care varies, and if a patient’s condition does not improve with advice and interventions from a general practitioner and/or exercises from a physiotherapist, a referral to an orthopaedic surgeon is often made [5, 6]. Orthopaedic waiting lists are often among the longest, and many patients on the lists do not require the specific surgical expertise and resources available in an orthopaedic outpatient department [7]; earlier studies have found that the number of patients in need of orthopedic surgery ranges from 30 to 68% [3, 8, 9]. When a patient finally has an appointment with an orthopaedic surgeon a wide range of interventions are often made, though not necessarily the most appropriate [10]. The prevailing system is often both time and resource consuming and causes prolonged suffering for patients, and there is a risk that even less severe conditions become persistent and more difficult to treat meanwhile being on the waiting lists**.** Providing correct diagnosis, appropriate care, and reducing waiting lists should be a societal priority, hence, it is important to try to identify new ways to offer effective care for orthopaedic outpatients.

According to current national guidelines in the UK (NICE 2020) [11] supported by a systematic review and meta-analysis [12, 13], large RCT:s [14, 15], and cost-effectiveness studies [15–17], the integration of manual therapy interventions for the management of patients with back and neck pain in conventional care may be feasible and cost-effective. In the Scandinavian countries the manual therapy profession naprapathy is very common. It is the most common profession within specialized manual therapy in Sweden (i.e. in this study meaning manual therapy professions with a 4–5 year specialization, licensed by the Swedish National Board of Health and Welfare). Nevertheless, health care decision makers, physicians and patients often don’t know exactly how manual therapy is defined, what naprapathy is and who is performing it, and therefore it seems difficult to consider an integration of it in mainstream care. Only a few percent of all licensed physiotherapists in Sweden are specialized in manual therapy, of which the majority work in the area of the capital city. Thus, it cannot be considered standard care for musculoskeletal disorders in the health care system in Sweden, or in other European countries. Moreover, there is still an overall shortage of evidence from studies of manual therapy interventions for other musculoskeletal pain than neck and low back pain in general, and with long term follow-up (i.e. more than 12 months) in particular. On orthopaedic waiting lists the pain location for the most common disorders are the upper and lower extremities.

In Great Britain, Canada and Australia specially trained physiotherapists known as "extended scope physiotherapists" have successfully acted as gate keepers, to reduce the load of orthopaedic surgeons, shorten the waiting lists [18, 19], reduce the waiting time for orthopaedic surgery [20], and compare diagnostic skills [21]. In a previously published, 12 months follow-up of a pragmatic randomized controlled trial (original research of Lilje et al. 2010) naprapathy improved bodily pain, physical function and health related quality of life (HRQoL) more than orthopaedic standard care, when treating working age orthopaedic outpatients. The health care costs for managing those common, non-surgical musculoskeletal disorders were also significantly lower when treated with naprapathy (original research of Lilje et al. 2014). The aim of the present study was to perform an 8-year follow-up (96 months) of the treatment effects of that trial, in order to increase the evidence of the effects of manual therapy.

## Materials and methods

### Design, setting and participants

The methods of the original trial/study are described in detail elsewhere (Lilje et al. 2010). In short, the trial initially randomized 98 working age patients in an orthopedic outpatient department of a region hospital in Blekinge county, in southern Sweden, for whom the most frequent location of complaints was the upper and lower extremities and the average pain duration was more than one year. The patients had been examined and received appropriate interventions in primary care (i.e. examination by a general practitioner and/or physiotherapy/radiography/injection/medication) before a referral to specialized care was made, and they were classified as non-surgical, thus low priority patients in the orthopaedic clinic [7]. The treatments lasted from January 2007 to November 2007. Demographic factors are shown in Table1, diagnostic codes in Table 2.

**Table 1:** Previous interventions and prognostic indicators at baseline in a pragmatic randomized controlled trial for non-surgical outpatients referred to orthopaedics

	Experimental group	Control group
	(n = 40)	(n = 38)
*Mean age, years*	38	45
Women %	42	60
*Location of the worst pain, %*		
Foot/leg	32	23
Shoulder/arm	20	19
Knee	13	18
Back	14	17
Elbow/hand	13	11
Head/neck	3	7
Pelvis/hip	5	5
*Duration of pain, %*		
< 3 months	5	5
3–12 months	30	29
> 12 months	65	66
*Earlier interventions, %*		
Doctor*	40	38
Physiotherapist	40	34
X-rays	50	55
Injection	20	18
Medicine†	52	45
Other‡	25	18
*Average pain*		
(VAS; 1–100: 100 = worst)	77	62
*Disability (ADL functions)*		
100%	4	4
75%	2	1
50%	7	4
25%	14	4
0%	13	25
*SF-36§*		
Pain	37.3	43.8
Physical function	70.4	73.3

* Apart from the referral consultation; GP, orthopaedist or emergency visit

^†^ Medicine requiring prescription only

^‡^ Chiropractor, osteopath, acupuncture, CRP/Borrelia/SR, orthosis, surgery

^§^ Higher value indicates less pain/better physical function

**Table 2:** Diagnostic Codes (ICD 10) documented by naprapath, respectively orthopaedist, at first visit in a pragmatic randomized controlled trial for non-surgical outpatients referred to orthopaedics

Location	Experimental group	Control group
*Neck*		
M530, M531, M542	1	2
*Shoulder/arm*		
M190, M191B, M244C, M294B,		
M653, M750, M751, M754, M770/771,		
M796B, S435, G560, G562C	13	11
*Back*		
M544, M545, M549, M626, Z039	5	7
*Pelvis/hip*		
M244	2	-
*Knee*		
M171, M222, M255, M626, M705,	5	7
S837, Z039		
*Leg/foot*		
M626, M628, M768/769, M201, M214	14	11
M242H, G576 M722, M766, M773,		
M775, M796H		
Summary	40	38

Patients were randomized to manual therapy (naprapathy; experimental group) or to conventional orthopaedic care (control group). The randomization was made pragmatically and subsequently by two nurses in the orthopaedic department at 5 different occasions, as soon as there was a number divisible by 2 eligible patients. Each pile of “low priority referrals” was put upside down, so that no information was visible, whereafter the first referral that was turned was labeled ‘1’, the second ‘2’, etc, resulting in one pile with referrals with even numbers, one with odd. The participants in the control group were coded with odd numbers and in the experimental group with even numbers, though the nurses were not aware of which numbers were assigned to which groups. The experimental group underwent 1–5 treatment sessions, during a maximum of 5 weeks. The treatments were patient centered, including massage, treatment of myofascial trigger points, manual mobilizations and/or manipulations, and electro therapy if required. All treatments were combined with a few, individually tailored home exercises. The control group followed standard clinical procedures (“standard care”), i.e. investigation with or without injections, tests, medication, referrals to radiography, physiotherapy, and/or other investigations, and/or re-referrals to an orthopaedic surgeon. For ethical reasons, and the fact that the trial was performed in a “real-world setting” where patients had been referred for orthopaedic consultation, cross-over from the experimental group was accepted. Patients in that group were allowed to have an appointment with an orthopaedic surgeon (cross over) after the 3 months follow-up (i.e. the protocol driven time of the trial) in case naprapathy had not been effective, or if a second opinion was warranted. In order to make the treatments in the control group as much “standard care” as possible and blinded for the orthopaedists, patients in the control group were not allowed to cross over.

The primary outcomes in the original trial were bodily pain, physical function and health related quality of life values (QoLs), measured at 3, 6 and 12 months. Secondary outcomes were patients’ perceived recovery, the total number of patients discharged from the orthopaedic waiting lists after manual treatment, the number of patients that crossed over, the level of agreement between orthopaedic and manual therapy diagnosis and management, and the number of health care interventions [7].

### Data collection for the 8-year follow-up

After 96 months (i.e. 8 years after baseline, and 7 years after the completion of the original 12 months follow-up) data were collected through postal questionnaires, patient records and telephone interviews. The present study reports on bodily pain and physical function (SF36), QoLs (SF6D), collected from postal questionnaire. The SF36 questionnaire is a validated instrument that contains 36 questions divided on 8 domains (i.e. general health, physical function, bodily pain, social function, physical role function, emotional role function, psychological wellbeing and vitality). Each item contains a score, with a higher score meaning a higher quality of life. When calculating QoLs the SF36 is encoded to the SF6D questionnaire, which is shortened to 6 domains, where 1 equals full health, and 0 equals death. In order to verify information on health care utilization (i.e. the number of health care visits both within the national health care system and alternative or complementary treatments) information was collected through patient records and telephone interviews. This was done by a nurse who was not aware of the participants’ group allocation.

### Statistical analysis

A power calculation was made in advance of the original study, to determine the sample size. A total of 80 patients indicated a power of 80% to detect a relative risk (RR) of 1.2 to 1.3 for a clinically important improvement in pain and physical function. At the 8-year follow-up bodily pain and physical function (SF36) were compared within and between the two groups using Wilcoxon’s signed rank test and Mann Whitney U test, respectively. The SF36 was converted to SF6D in order to calculate QoLs, calculated as individual mean values with accompanying confidence intervals (95% CI). *P*-values were considered significant at < 0.05. Information about health care utilization was collected as descriptive data and a sensitivity analysis using the same statistics as above was made in which all alternative treatments were withdrawn. An intention to treat analysis was used with participants analyzed in the assigned groups.

## Results

### Summary of previous results at 12 months follow-up

The participants’ most common locations of pain mirrored the proportions on the waiting lists as a whole, with regard to age, gender and pain locations (the upper and lower extremities being the most common). Statistically significant differences between the groups were found as regards improvement in bodily pain, physical function and perceived recovery, in favor of the experimental group. The total number of health care interventions was 275 (of which 46 were self-elected) in the experimental group compared with 379 (of which 33 were self-elected) in the control group [7]*.* Sixty-two percent of the patients in the experimental group agreed to be discharged from the waiting lists at the 12 months follow-up, and the level of diagnostic agreement between manual therapist (naprapathy) and orthopaedist with regard to the patients that crossed over was 80%. When the outcomes of the cross-over patients in the experimental group were compared with the rest of their group, after their additional orthopaedic consultation, they did not differ from each other.

### Summary of 96 months follow-up

**Fig. 1:**
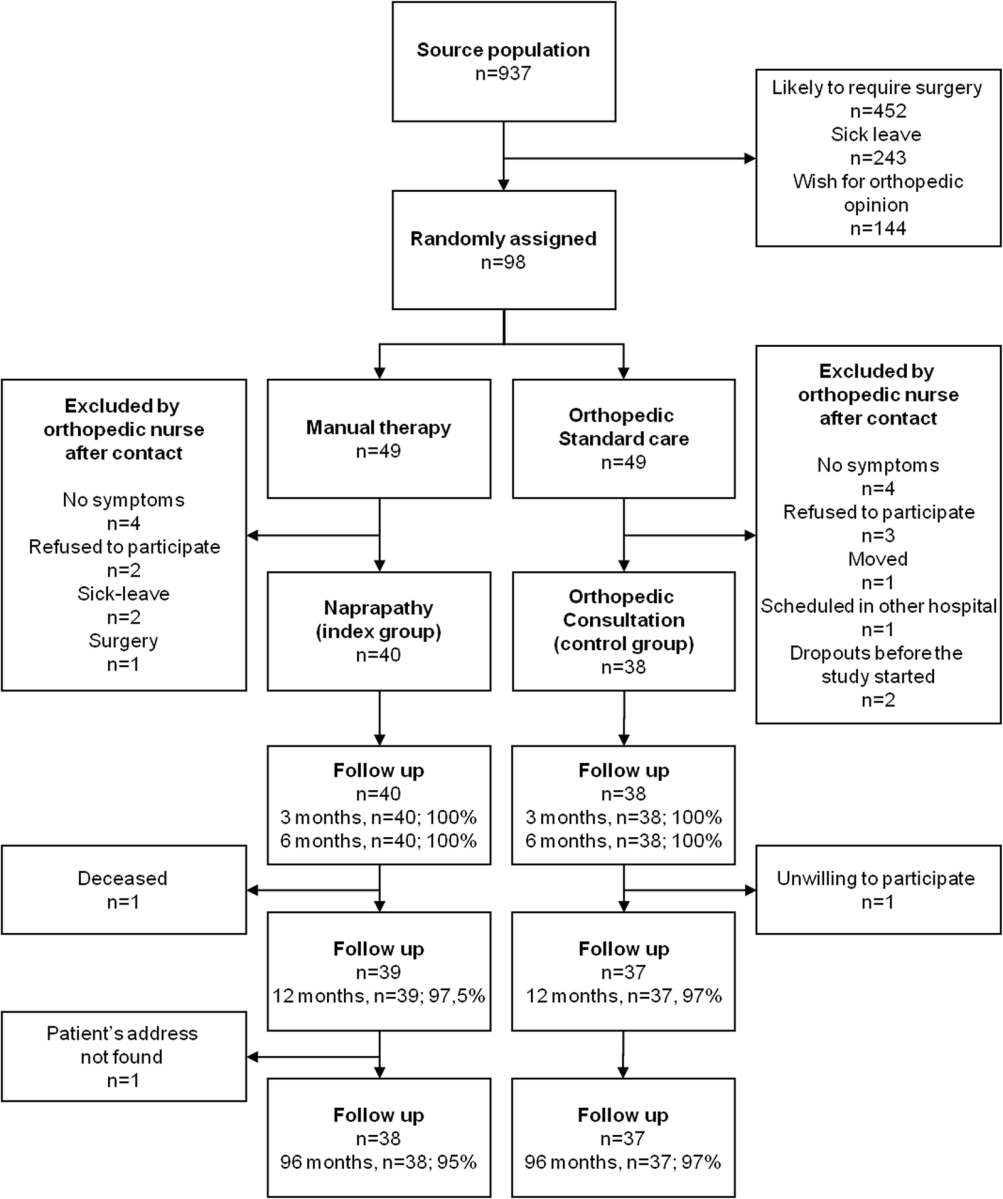
Flow chart describing the progress of patients throughout the pragmatic randomized controlled trial

Altogether ninety-eight patients were randomized, of which 78 patients participated in the trial. Seventy-six (96%) of the 78 patients participating in the pragmatic randomized controlled trial on non-surgical orthopaedic outpatients were followed up after 8 years. For the participants’ progress through the trial see Fig. 1.

The number of health care interventions were significantly lower in the experimental group, as seen in Table 3.

**Table 3:** Healthcare interventions for the participants of a pragmatic randomized controlled trial on orthopaedic outpatient disorders, respectively at 1 and 8 year follow-up

Type of intervention	Number of interventions at 12 months (1 year)		Number of interventions at 96 months (8 years)	
	Control group (n = 38)	Experimental group (n = 40)	Control group (n = 37)	Experimental group (n = 38)
Naprapathy	N.a	166 (40)	13 (2)	4 (2)
Orthopedics	53 (38)	15 (15)*	8 (4)	0
Physiotherapy	242 (13)	31 (2)*	773 (11)	21 (3)
Orthotics	6 (6)	1 (1)*	12 (1)	1 (1)
General practitioner	0	0	7 (2)	0
Radiography/tests	20 (19)	12 (6)*	0	0
Surgical procedures	7 (7)	1 (1)**	2 (2)	5 (5)
Drugs/injections	18 (18)	3 (3)*	0	0
Alternative treatments***	33 (5)	46 (5)	668 (7)	4 (1)
Total number of treatments:	379	275	1 483	35

The figures in brackets indicate the number of participants receiving each treatment. Some participants had more than one intervention made. The data from baseline—12 months have been published earlier [7].

N.a. = Not applicable.

*Cross-over patients from the experimental group (i.e. after the 3 months protocol driven follow-up time of the RCT).

**One of the 4 patients referred to surgery in the manual experimental group underwent surgery.

***Alternative treatments: chiropractic, massage, water exercises, lymphatic massage, personal training, shockwave.

**Fig. 2:**
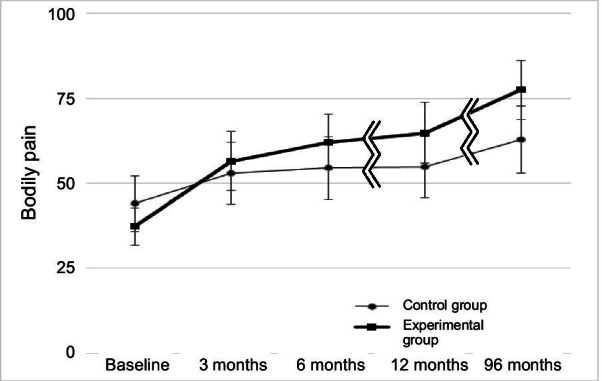
Bodily pain (SF36) for the experimental and the control group at follow-up 3, 6, 12 and 96 months after baseline

**Fig. 3:**
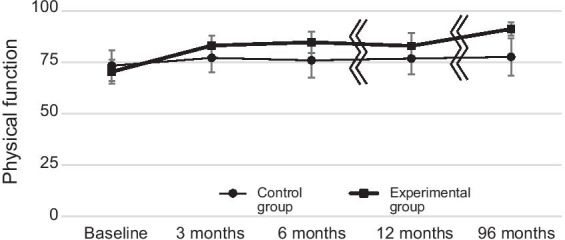
Physical function (SF36) for the experimental and the control group at follow-up 3, 6, 12 and 96 months after baseline

There were statistically significant differences in bodily pain in favor of the experimental group: 39.9 (95% CI: 30.9–48.8) compared with 18.2 (95% CI: 9.0–27.4); difference in change: 21.7 (95% CI: 9.1–34.3), *p*-value < 0.01 (Table 4, Fig. 2), for physical function: 21.1 (95% CI: 6.9–34.4) for the experimental group compared with 3.5 (95% CI: − 5.2–12.2) for the control group; difference in change: 17.6 (95% CI: 6.7–28.4), *p*-value < 0.01 (Table 4, Fig. 3), and for HRQoL; 0.823 (95% CI: 0.785–0.862) for the experimental group compared with 0.713 (0.668–0.758) for the control group, *p*-value < 0.01 (Table 5).

**Table 4:** The difference in changes in bodily pain (BP) and physical function (PF) between the manual therapy and the control group between baseline, 3, 6, 12 and 96 months

	Baseline	3	Months	6	Months	12	Months	96 months	(8 years)
	Baseline value	Change	Difference in change	Change	Difference in change	Change	Difference in Change	Change	Difference in change
	(95% CI)	(95% CI)	(95% CI)	(95% CI)	(95% CI)	(95% CI)	(95% CI)	(95% CI)	(95% CI)
*BP*			*P* = 0.043		*P* = 0.015		*P* = 0.003		*P* < 0.001
Manual therapy group	37.3 (31.7–4.7)	19.2 (1.2–26.4)	10.2 (0.9–19.4)	24.9 (17.4–32.3)	14.0 (4.0–23.9)	28.2 (20.8–35.7)	17.6 (7.3–27.9)	39.9 (30.9–48.8)	21.7 (9.1–34.3)
Control group	43.8 (35.6–52.0)	9.0 (3.0–15.1)		10.9 (4.1–17.7)		10.6 (3.3–18.0)		18.2 (9.0–27.4)	
*PF*			*P* = 0.003		*P* = 0.005		*P* = 0.002		*P* < 0.001
Manual therapy group	70.4 (64.5–76.3)	12.8 (6.8–18.8)	9.0 (1.6–16.3)	14.4 (8.5–20.4)	11.5 (3.5–19.6)	13.1 (5.9–20.2)	10.2 (1.9–18.4)	21.1 (6.9–34.4)	17.6 (6.7–28.4)
Control group	73.3 (65.9–80.8)	3.8 (− 0.5–8.1)		2.9 (− 2.7–8.4)		2.9 (− 1.5–7.3)		3.5 (− 5.2–12.2)	

*P*-values correspond to the difference in changes between the groups

Higher scores for BP indicates improvement in pain, higher scores for PF indicates improved physical function. The maximum (most preferrable) score for BP and PF is 100

**Table 5:** QoL scores at baseline, 3, 6, 12 and 96 months for the experimental and the control group

Group	Month	Sum	Number of participants	Mean value/participant with 95% CI
Control group	0	26.108	38	0.687 (0.636–0.738)
	3	26.417	38	0.695 (0.647–0.743)
	6	27.532	38	0.725 (0.675–0.774)
	12	26.909	37	0.727 (0.675–0.779)
	96	26.372	37	0.713 (0.668–0.758)
Experimental group	0	26.225	40	0.656 (0.620–0.691)
	3	29.490	40	0.737 (0.694–0.780)
	6	30.154	40	0.754 (0.707–0.800)
	12	29.282	38	0.771 (0.722–0.819)
	96	31.288	38	0.823 (0.785–0.862)

Confidence intervals 95%, *p*-values < 0.01

The maximum, most preferrable score of QoL is 1.0

The experimental group had a total of 310 health care visits compared with 1 862 in the control group, as seen in Table 3. When withdrawing all alternative treatments in the sensitivity analysis, the number of visits in the experimental group was 260 and 1161 in the control group. All in all, during the whole study period from baseline to 8 years, there were 6 participants in the experimental group that underwent surgery compared with 9 participants in the control group. The number of patients in the experimental group who had continuing care after 12 months was n = 2, and after 96 months n = 0 compared with n = 12 and n = 6, respectively, in the control group. Fifteen patients in the experimental group also had an appointment with an orthopaedist, i.e. became cross-overs. Four of those were candidates for surgery (n = 2), required orthopaedic intervention, and the remaining patients (n = 9) had improved, but wanted an orthopaedic assessment/second opinion, which was allowed. No differences were found when analyzing the patients that crossed over separately and compared with the results of the rest of the experimental group.

## Discussion

### Summary of findings

In summary the results showed that patients who received naprapathy had larger improvements in pain, physical function, quality of life, and fewer healthcare visits compared with the control group, thus, the results are consistent with those from the 12-months follow-up of the original study [7]. The control group’s physical function was more or less unchanged for the whole follow-up period of 8 years, and the improvement in bodily pain was smaller in the control group compared with the experimental group. Meanwhile, the total number of health care visits was significantly larger in the control group. Physiotherapy is usually a part of standard care for non-surgical orthopaedic outpatients both in primary and secondary, specialized health care, and it was by far the most common intervention in the control group at the 12-months as well as at the 96-months follow-up. The physiotherapy employed in the present study was general physiotherapy (i.e. rehabilitation through physical exercises) [22]*.* To the best of our knowledge this is the first trial on the effects of a manual therapy compared with standard orthopaedic care for the most common disorders in low priority non-surgical outpatients in specialized care, with longer follow-up than 12 months*,* and therefore there are no related articles in the published literature for direct comparison with our study.

### Strengths and weaknesses/methodological considerations

Strengths of our study include the research question, the study design, with “real world data” from a clinical setting, a high response rate even at the 8-year follow up (i.e. 96%), and the fact that all outcomes were significantly better in the experimental group. The location of the disorders, age and gender closely mirrored orthopaedic waiting lists in general, which strengthens the study’s external validity. The outcomes in pain, physical function and health care utilization were also consistent with those between baseline, 3, 6 and 12 months in the original study in terms of differences between the groups. Though the differences between the groups for all outcomes were even larger between 12 and 96 months compared with earlier follow-ups. This is in contrast to earlier trials on other manual therapy or physiotherapy for musculoskeletal disorders, where follow-ups were only made until 12 months, and the initial differences in effects by then had lost statistical significance [15, 23, 24]. Pain, physical function and QoLs were derived from validated instruments (the SF36 and SF6D, respectively) and information about the participants’ health care utilization comprising both alternative care and traditional care within the national health care system were included, all of which we consider as strengths*.* During the first 12 months follow-up in the original trial the number of alternative treatments was low, and equally distributed in both groups. This suggests that it did not contribute to bias with regard to the outcomes of the original study, which increases the study’s internal validity. At follow-up after 96 months the number of alternative treatments increased, why a sensitivity analysis regarding those treatments was performed, which is also a strength. The study also has weaknesses that need to be taken into account. The initial power calculation suggested a sample size of 80 participants, and at the 8-year follow-up the number of participants was n = 75 (i.e. 96%), which may have resulted in an underpowered study. However, given the long follow-up period, and the fact that the outcomes were consistent, it is unlikely that a loss of only 5 participants would have changed the conclusions of our study. The fact that the manual therapy in the experimental group was performed in the shape of naprapathy may be seen as a weakness when considering the generalizability of the results, but the manual techniques used by naprapaths are very similar to those given by other manual therapists such as chiropractors and osteopaths, which makes it possible to generalize the results. Another weakness is that no follow-ups were made between 12 and 96 months (8 years). It would have been interesting to know more about the participants’ health status during the study period, though this was not possible due to reasons of feasibility and lack of resources. Nevertheless, the outcomes were consistent and clinically relevant at all follow-ups, why it is not sure that more frequent follow-ups would have had an impact on the conclusions of our study. More frequent follow-up periods might provide different and more detailed perspectives of clinical and cost related consequences over time though, which should be considered in future studies. The information about health care utilization was collected retrospectively, which may also be considered a weakness. The data were recorded over telephone and cross checked in the hospital’s information system, and this was done in order to minimize the risk of recall bias and misunderstandings regarding health care utilization and alternative treatments. A large amount of alternative treatments were used by a few participants in the control group between 12 and 96 months which may be considered a weakness, though sensitivity analyses showed that even after excluding all alternative treatments from the analyses there were still statistically significant differences between the groups as regards health care utilization. More surgical interventions were made in the experimental group (n = 5) between 12 and 96 months, compared with the control group (n = 2). However, this was due to the fact that 3 of the surgical interventions in the experimental group had been postponed from the original trial (i.e. participants assessed as surgical cases chose not to have surgery at that time). Moreover, the total number of surgical interventions for the whole follow-up period from baseline to 8 years was higher in the control group compared with the experimental group (n = 9 compared with n = 6).

### Manual therapy

Earlier research has shown that combining one or more treatment techniques with home exercises, like within the pragmatic approach used in naprapathy has proved to be effective [25] and the results of our study are in line with earlier performed research on naprapathy and chiropractic for neck and back pain, and other musculoskeletal disorders, with 12 months follow-up [14, 26–29]. Other than the effectiveness of combining different treatment techniques another possible explanation for the larger improvement in the experimental therapy group in the present study may be the patient centered care and patient empowerment approach [30] used in naprapathy. The participants are active during the treatment sessions and after, through individualized home exercises [31], which may be the reason why the experimental group kept improving, in particular in physical function. A typical manual therapy treatment session is longer than that of an orthopedic consultation (i.e. 30–45 min, as compared to 10–20 min), which possibly also allows for the patient to reflect and to ask all necessary questions. The exercises given in the experimental group were patient centered; pragmatic and adjusted to the patients’ individual needs during the treatment period, and it is plausible that this contributed to patient empowerment and positive copying strategies, believed to have an impact on treatment outcomes [30]. There are a lot of shorter educations in different general manual therapies, and there are professions specialized in manual therapy, for example naprapaths, chiropractors, osteopaths, and physiotherapists with an extended education in Orthopedic Manual Therapy (OMT), and in Sweden and Scandinavia all of these professions—except osteopathy—are currently licensed by the National Board of Health and Welfare. There are similarities between specialized manual therapy (naprapaths, chiropractors, osteopaths and OMT physiotherapists) and general physiotherapy, and there are also key differences as regards the lengths and contents of the different educations [22, 32]. Just like in for example Canada, Australia, and the Netherlands, an additional post graduate training is required for physiotherapists in order to be specialized in manual therapy (i.e. OMT), that is, educated in different mobilization techniques and high velocity, low amplitude manipulations. Approximately 2% of all licensed physiotherapists in Sweden have this specialization [33].

### Physiotherapy

Earlier studies on orthopaedic outpatients have investigated the role of physiotherapy in order to reduce waiting lists and found that extended scope physiotherapists are effective in triaging patients on the waiting lists for orthopaedic consultation [19–21]. The focus of these studies were on a physiotherapeutic assessment of patients, not the effectiveness of physiotherapy per se, and only one of these studies had a randomized controlled design [34]. That study showed differences in favor of the physiotherapy decisions in terms of HRQoL, pain-related disability and sick leave at 3 months but lost statistical significance at 12 months [24]. The aim of the present study was to compare a specialized manual therapy with standard orthopaedic care, though standard care for low priority orthopaedic outpatients with non-surgical musculoskeletal disorders most often consists of general physiotherapy, which was the far most common intervention in the control group. The participants in that group received a large number of physiotherapy sessions until the 12-months follow-up (altogether 242 sessions for 1/3 of the control group compared with 166 naprapathic sessions for the whole manual therapy group), still, the experimental group improved more, and at lower costs [10].

General physiotherapists work with “hands-off treatment” aimed to prevent, treat and rehabilitate musculoskeletal pain and disability mainly through patient movements, advice on ergonomics, and physical exercises [22, 35]. Specialized manual therapists work to prevent, diagnose and treat musculoskeletal pain and disability using “hands-on treatment” such as massage and different mobilization and/or manipulation techniques, combined with physical home exercises [36].

Resource utilization is applied when taking care of musculoskeletal pain and disorders in national health care systems. That was a less effective way to manage the disorders of the sample of secondary health care patients in the present study compared with the complementary therapy naprapathy*.* Large high quality trials on the cost effectiveness of specialized manual therapy for all kinds of musculoskeletal disorders and follow-ups of more than 12 months are warranted, in order for policy makers to facilitate adequate evidence-based decisions regarding appropriate triaging of patients with musculoskeletal disorders [37].

## Conclusions

In conclusion, naprapathy yielded significantly better long-term improvement and fewer health care interventions than orthopaedic standard care for the most common non-surgical musculoskeletal disorders in working age outpatients in specialized care. Together with the outcomes from previously published studies the results suggest that specialized manual therapy is an effective treatment that should be considered when triaging patients with common non-surgical musculoskeletal disorders in national health care systems.

## Data Availability

The datasets generated and/or analysed during the current study are not publicly available due to data protection of participants but are available from the corresponding author on reasonable request.
